# Nei Endonuclease VIII-like 2 Gene rs8191670 Polymorphism affects the Sensitivity of Non-small Cell Lung Cancer to Cisplatin by binding with MiR-548a

**DOI:** 10.7150/jca.47495

**Published:** 2020-06-06

**Authors:** Wei He, Lina Pang, Shuai Gong, Xin Wang, Lixia Hou

**Affiliations:** 1 Department of Oncology, The First Affiliated Hospital of Zhengzhou University, Zhengzhou, Henan 450052, P.R. China.; 2 Department of Radiotherapy, The First Affiliated Hospital of Zhengzhou University, Zhengzhou, Henan 450052, P.R. China.

**Keywords:** NEIL2, rs8191670, single nucleotide polymorphism, miR-548a, NSCLC, chemotherapy resistance

## Abstract

**Background:** Nei endonuclease VIII-like 2 (NEIL2) is a gene encoding DNA repair enzyme, which is involved in the base excision repair (BER) pathway in mammalian cells. Cisplatin is a common cytotoxic anti-tumor agent in clinic by destroying normal structure of DNA and inducing cell apoptosis. However, how NEIL2 affects the sensitivity of NSCLC to cisplatin is still unclear.

**Methods:** The clinical data from 206 patients diagnosed pathologically were collected. The DNA sequencing of *NEIL2* gene 3'UTR and the PFS curve of NSCLC patients receiving cisplatin-based chemotherapy were performed. Western blot analysis and immunohistochemistry were used to detect NEIL2 protein expression. Human NSCLC cell lines A549 and H1299 were cultured and evaluated for cell viability. RT-PCR was performed for quantitative detection of miR-548a. 3′UTR reporter plasmid was constructed and luciferase reporter assay was used to verify the target gene regulated by miR-548a.

**Results:** In this study, we found that the *Neil2* gene had the polymorphism (T/C) in rs8191670 and it is associated with the PFS of advanced NSCLC patients. MiR-548a targets *NEIL2* 3'UTR to suppress its expression. Upregulation of NEIL2 expression or downregulation of miR-548a could reduce the sensitivity of NSCLC cells to cisplatin.

**Conclusion:** Our results demonstrated that NEIL2 gene rs8191670 polymorphism affects the PFS of advanced NSCLC patients, and the underlying molecular mechanisms may be that miR-548a can regulate NEIL2 expression by binding to its 3'UTR seed region containing rs8191670.

## Introduction

Lung cancer is the most frequently diagnosed cancer up to now throughout the world 1. More than 80% of lung cancers are non-small cell lung cancer (NSCLC), and more than half of which were diagnosed at advanced or locally advanced stage 2. Although great progress has been made in the treatment of NSCLC, such as immunotherapy and molecular targeted therapy, the 5-year survival rate for lung cancer is still less than 15% 3-4. Cisplatin-based combination chemotherapy is still the first-line treatment for advanced NSCLC without driver gene, but the response rate was fluctuating between 26% and 60% 5. Therefore, it is very urgent and crucial to investigate the underlying molecular mechanism and look for novel potential therapeutic targets for the treatment of NSCLC.

Cisplatin is a cell cycle non-specific cytotoxic anti-tumor agent, which form cisplatin-DNA adduct when it enters tumor cells. DNA cross-linking is formed by linking DNA-pt-DNA structure, which leads to destroy the normal structure of DNA, inhibit DNA replication and transcription, and induce cell apoptosis. For the last 30 years, cisplatin has been the most widely prescribed chemotherapy drug for treating a variety of cancers, especially lung cancer, ovary cancer, and esophageal cancer. Cisplatin is often effective in the beginning, but the major drawback is the development of chemo-resistance through treatment. The main mechanism of cisplatin resistance is the defective of DNA damage repair function 6. It is of great significance to explore the resistance mechanism of platinum-based agents in order to improve the effect of chemotherapy and the survival rate of cancer patients.

Nei endonuclease VIII-like 2 (NEIL2), a member of endonuclease VIII family, is a gene encoding DNA repair enzyme, and is mainly involved in the base excision repair (BER) pathway in mammalian cells 7-8. The BER pathway is the most active DNA damage repair pathway in mammals, which plays an important role in maintaining the stability of genome and inhibiting tumor development. It plays a positive protective role in DNA damage caused by ultraviolet radiation, alkylation agents and oxidative stress 9-10. In recent years, some studies have shown that NEIL2 is highly expressed in a variety of cancers 11. The ability to repair DNA damage caused by platinum-based chemotherapy was enhanced by increased intracellular NEIL2 protein, which leads to tumor resistance to platinum-based chemotherapy. Therefore, NEIL2 may be a potential drug target for tumor chemosensitivity, which provides a new prospect for chemotherapy in patients and opens a new field for improving the prognosis of patients with NSCLC. However, very few studies have explored the relationship between NEIL2 and chemotherapy sensitivity in NSCLC.

MicroRNAs (miRNAs) are short RNA molecules that negatively regulate gene expression primarily by degrading target mRNA or inhibit the translation of target mRNA into protein. Recently, many reports have shown the altered miRNA expression in various diseases 12-13, which is also ubiquitous in many tumor tissues, such as gastric cancer and pancreatic cancer. Moreover, specific circulating miRNA expression profiles have been found in patients with lymphoma, leukemia, gastric cancer, lung cancer, breast cancer and other cancers 14-17. The relationship between NEIL2 and NSCLC is largely unknown. Our study investigates the role of NEIL2 in NSCLC chemoresistance and how NEIL2 is regulated.

## Materials and methods

### Patients and clinical data

Clinical data in this study was obtained from 206 NSCLC patients in the First Affiliated Hospital of Zhengzhou University from May 2011 to January 2017 (Supplementary Table 1). 2 mL of peripheral blood from these patients were collected. All patients were newly diagnosed stage IV NSCLC patients without any previous treatment. All specimens were collected after obtained the informed consents from the patients. This study was also approved by the Ethics Committee of the First Affiliated Hospital of Zhengzhou University.

Baseline data was collected from the date when the patient was pathologically diagnosed with NSCLC until the date that computed tomography (CT) examination showed the disease progress or the patient died. The follow-up visits were scheduled every 3 months until March 2017. The follow-up time was from 3 to 57 months, and the median follow-up time was 6.4 months. There were no lost follow-up patients. The follow-up rate for mortality was 100%. Survival analysis was performed based on follow-up data.

### Detection of NEIL2 gene polymorphism

DNA sequencing was used to detect mutations in the 3′-untranslated region (3'-UTR) of NEIL2 gene in peripheral blood of NSCLC patients. Genomic DNA was extracted using whole blood genome DNA extraction kit from BioTeke Corporation (Wuxi, CHN) according to the recommended protocol. Based on the NEIL2 gene sequence (NM_001135746) published on GenBank, Primer Premier 5 software was used to design PCR primers. The PCR specific primers are 5'-TCATCCTGTTGAATTGCACCA-3' (forward) and 5'-GGTGGCTCACACCCGTGGTCCCAA-3' (reverse) for NEIL2 gene. PCR was performed using genomic DNA as a template. The PCR reaction system was 18 μL of H_2_O, 3 μL of Buffer, 2 μL of dNTP, 1 μL of Taq enzyme, 0.5 μL each of upstream and downstream primers, and 5 μL of genomic DNA. The conditions as shown below: 95˚C for 2 min, followed by 95˚C for 30 sec, 58˚C for 35 sec, 72˚C for 40 sec, 35 cycles in total; and at last, 72˚C for 3 min. 2% agarose gel electrophoresis was performed to identify the PCR products. The length of the target gene NEIL2 3'-UTR is 450 bp. DNA sequencing were used to detect PCR products from gel extraction. All samples were sequenced by Biotech Bioengineering (Shanghai) Co., Ltd. The single nucleotide polymorphism (SNP) in rs8191670 of *NEIL2* gene was analyzed too.

### Cell culture, transfection and reagents

Two NSCLC cell lines (A549, H1299) and the human embryonic kidney cell line (HEK293T) were purchased from the Cell Bank of the Chinese Academy of Medical Science. Both NSCLC cell lines were cultured in RPMI 1640 medium (HyClone, USA) supplemented with 10% fetal bovine serum (HyClone, USA) and 1% penicillin/streptomycin (Invitrogen, USA) at 37 °C under 5% CO_2_ and saturated moisture. HEK293T cells were cultured in DMEM/high glucose medium (Hyclone, USA) supplemented with 10% fetal bovine serum (HyClone, USA) and 1% penicillin/streptomycin (Invitrogen, USA) at 37 °C under 5% CO_2_ and saturated moisture. MiR-548a mimics, miR-548a inhibitor or their negative controls (miR-scramble, Inhibitor-NC) (GenePharma, CHN) was transfected transiently into NSCLC cell lines using Lipofectamine 2000 (Invitrogen, USA) according to the manufacturer's instructions. The transfected amount of miRNA was 10 pmol per 1 × 10^3^ cells.

The primary antibody against NEIL2 (Catalog No. PA5-84913) was obtained from Invitrogen (California, USA). And β-actin (Catalog No. sc-47778) was obtained from Santa Cruz Biotechnology (Santa Cruz, CA). Cisplatin was purchased from Selleck Chemicals (Houston, TX, USA).

### Real-Time PCR analysis

For quantitative detection of miR-548a, qRT-PCR analysis was performed using the Two Step Stemaim-it miR-548a qRT-PCR Quantitation Kit (Novland, China). We quantified U6 small nuclear RNA (U6 snRNA) as an endogenous control to normalize miRNA level. Each sample was analyzed in triplicate on the ABI7500 Fast thermocycler (Applied Biosystems, USA).

### Immunohistochemistry

Immunohistochemical analysis for NEIL2 was performed on 4-μm sections. The Envision Plus detection system (Dako, USA) was used for the detection of immunostaining. Tissue sections were pretreated with 10 mM sodium citrate buffer for antigen unmasking (pH 6.0) after deparaffinized in xylene. Endogenous peroxidase activity was blocked by incubation with 0.03% hydrogen peroxide in methanol for 15 min. Then sections were incubated with primary antibodies at 4°C overnight after blocked in normal serum for 30min. Next, Sections were incubated with secondary antibody at room temperature for 60 min before staining for 5 min with 3'3-diaminobenzidine tetrahydrochloride, counterstained by hematoxylin, dehydrated, and mounted in Diatex. Quantitative analysis of IHC staining was performed using the Image-Pro Plus software (v.6.0) program (Media Cybernetics, Inc., USA).

### Construction of 3′UTR reporter Plasmid and Luciferase assay

The 3′UTR of *NEIL2*, which contains a putative target region for miR-548a, was PCR amplified from genomic DNA with primers 5'-GAACTCGAGAAGGCAGAGTTTTCATAGGGTTAGA-3' (sense) and 5'-CACTCTAGACATGCCTGTAGTCCCAGCTACTCTG-3' (antisense). The NEIL2 3′UTR with SNP “C” construct was PCR amplified by using the genomic DNA as template from patient with NEIL2 rs8191670 “C/C”. The NEIL2 3′UTR mutant construct was generated by overlap extension PCR. Fragments were inserted between the *Xho*I and *Xba*I sites in the pmirGLO (Promega, USA). HEK293T cells were seeded in 96-well plates at a density of 1 × 10^4^ cells per well for 24 h before co-transfection. Co-transfection of the reporter vector (pmirGLO-wt-NEIL2 or pmirGLO-mut-NEIL2) and miRNA (miR-548a mimics or scramble) was performed using Lipofectamine 2000 (Invitrogen, USA). 24 hours after transfection, firefly and renilla luciferase activities were measured using the Dual Luciferase Reporter Assay Kit (Promega, USA) according to the manufacturer's protocol.

### Lentiviral infection

The lentiviral particles harboring the full-length cDNA sequence of exogenous NEIL2 (EX-NEIL2) or negative control (NC) (Vigene Corporation, China) were combined with 8 μg/ml of polybrane (Millipore, USA) and infected overnight into 60% confluent A549 and H1299 cells. The cell culture medium was replaced with fresh complete growth medium and after 24 hours, cells were selected with 2 μg/ml of puromycin for an additional 24 hours. The selected cells were used for the following analysis.

### Cell viability assay

The effect of miR-548a on the proliferation of the NSCLC cell lines was evaluated by the MTT assay. A549 and H1299 cells were plated in 96-well culture plates (2 × 10^3^ per well) and transfected with 30 pmol of miR-548a mimics or scramble for 24 hours, then treated with cisplatin (0, 1, 2, 5, 10, 20, 30 µM) for 48 hours. Then the cells were incubated with 100 μl 0.5 mg/ml MTT (Sigma-Aldrich, USA) at 37 °C for 4 hours, and the precipitate was dissolved in 150 μl dimethylsulfoxide (DMSO). After mixed for 10 minutes, the optical density at 570 nm was measured and the IC50 value was calculated on the non-linear regression fit method by Graphpad Prism 5.0 software (San Diego, CA). Each experiment was performed in triplicate.

### Western blotting

The cells after treated with reagents were washed in PBS and lysed with RIPAlysis buffer (CWBIO, CHN). Protein concentrations were determined by BCA protein assay kit (Thermo, USA). The samples corresponding to 20μg of protein were resolved on an 8%-15% denatured SDS polyacrylamide gel, and transferred onto a PVDF membrane (Millipore, USA). 5% skim milk was used to block non-specific binding sites for 1 hour, and then the membranes were probed with specific primary antibodies overnight at 4 °C. Next, the membranes were washed 3 times with TBS-Tween 20 followed by incubation with a horseradish peroxidase (HRP)-conjugated secondary antibody for 1 hour at room temperature. The protein bands were visualized with an Immobilon Western Chemiluminescent HRP Substrate (Millipore, USA). Image J software was used to analyze the expression of each protein, which was normalized by β-actin. Western blotting assay was repeated at least three times on every sample with similar results.

### Statistical analysis

The relationship between rs8191670 (T/C) polymorphism of NEIL2 and survival rate of NSCLC patients was analyzed by Kaplan-Meier methods, and the 95% confidence interval (CI) was calculated by log-rank analysis to analyze the difference between groups. Results were shown as mean values ± s.d. Statistical analysis was performed by Student's t-test, or one-way ANOVA. A level of P<0.05 was considered to be significant.

## Results

### rs8191670 polymorphism in *NEIL2* gene is associated with mPFS of NSCLC patients

**Figure 1:**
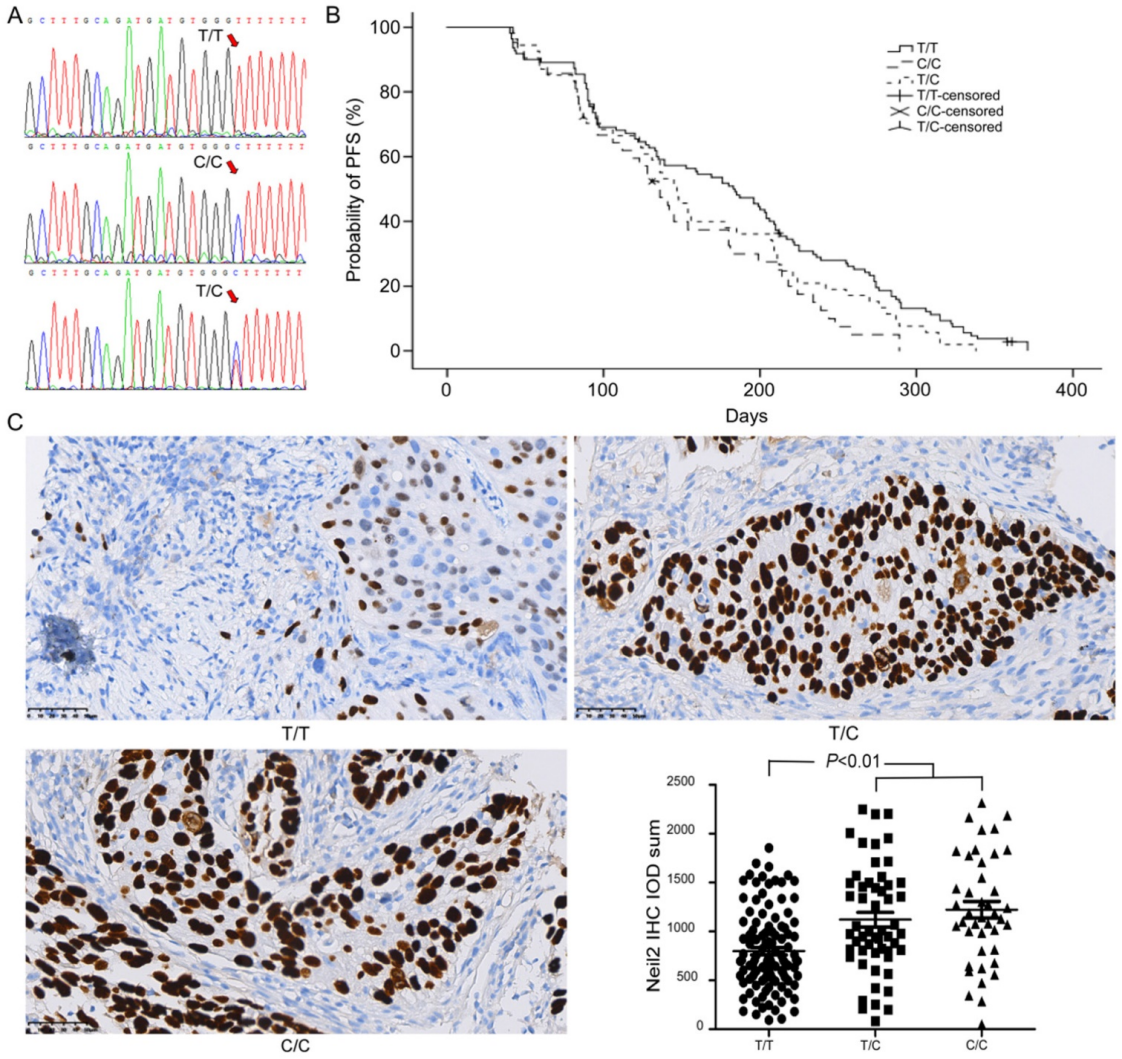
***NEIL2* gene rs8191670 polymorphism affects PFS of advanced NSCLC patients. A:** The sequencing result of rs8191670 polymorphism in *NEIL2* gene. **B:** The PFS curves of advanced NSCLC patients with different *NEIL2* rs8191670 polymorphism (N=206). The median mPFS of NSCLC patients bearing “T/T”, “T/C”, and “C/C” homozygote in *NEIL2* gene rs8191670 locus were 6.1m, 4.9m, and 4.5m, respectively. The difference between “T/T” and “C/C” groups was statistically significant (*P* = 0.01). **C:** The expression of NEIL2 in NSCLC with different *NEIL2* rs8191670 polymorphism.

DNA sequencing analysis showed that T/C polymorphism was found in rs8191670 locus of *NEIL2* gene (Figure 1A). Among 206 NSCLC patients, there were 110 “T/T” homozygote cases (53.4%), 42 “C/C” homozygote cases (20.4%) and 54 “T/C” heterozygote cases (26.2%).

After cisplatin-based chemotherapy, the median progression-free survival (mPFS) time of NSCLC patients bearing “T/T” homozygote in *NEIL2* gene rs8191670 locus was 6.1 months (95% CI: 5.0 months - 7.2 months), which was significantly longer than that of the “C/C” homozygote patients (4.5 months, 95% CI: 3.8 months - 5.2 months, *P* = 0.01). The mPFS of “T/C” heterozygous patients was 4.9 months (95% CI: 4.3 months - 5.5 months), the difference between “T/T” homozygote and “T/C” heterozygote was not statistically significant (*P* = 0.095, Figure 1B). The difference between “C/C” homozygote and “T/C” heterozygote was also not statistically significant, (*P* = 0.24, Figure 1B). In other words, T/C polymorphism in rs8191670 locus of *NEIL2* gene was associated with mPFS of NSCLC patients receiving cisplatin-based chemotherapy.

The chemotherapy adverse reactions of 206 patients were detailed in the Supplementary Material. Briefly, there is no significant difference in the incidence of adverse reactions (including nausea, vomiting, diarrhea, leukopenia, anemia, thrombocytopenia, and fever related leukopenia) among the three genotypes of patients during chemotherapy treatment (Supplementary Table 2).

### The expression of NEIL2 protein in NSCLC patients with different NEIL2 rs8191670 polymorphism

In order to explore the correlation between different polymorphism genotypes of *NEIL2* and their protein express, the expression level of NEIL2 proteins in NSCLC tissues was analyzed by immunohistochemistry staining. Our results showed that the expression level of NEIL2 protein was significantly higher in NSCLC patients bearing “C/C” homozygous and “T/C” heterozygous than in “T/T” homozygote patients (*P* < 0.01; Figure 1C). Therefore, the polymorphism in rs8191670 of *NEIL2* gene could affect the expression level of NEIL2 protein.

### miR-548a targets *NEIL2* 3'UTR to suppress its expression

**Figure 2:**
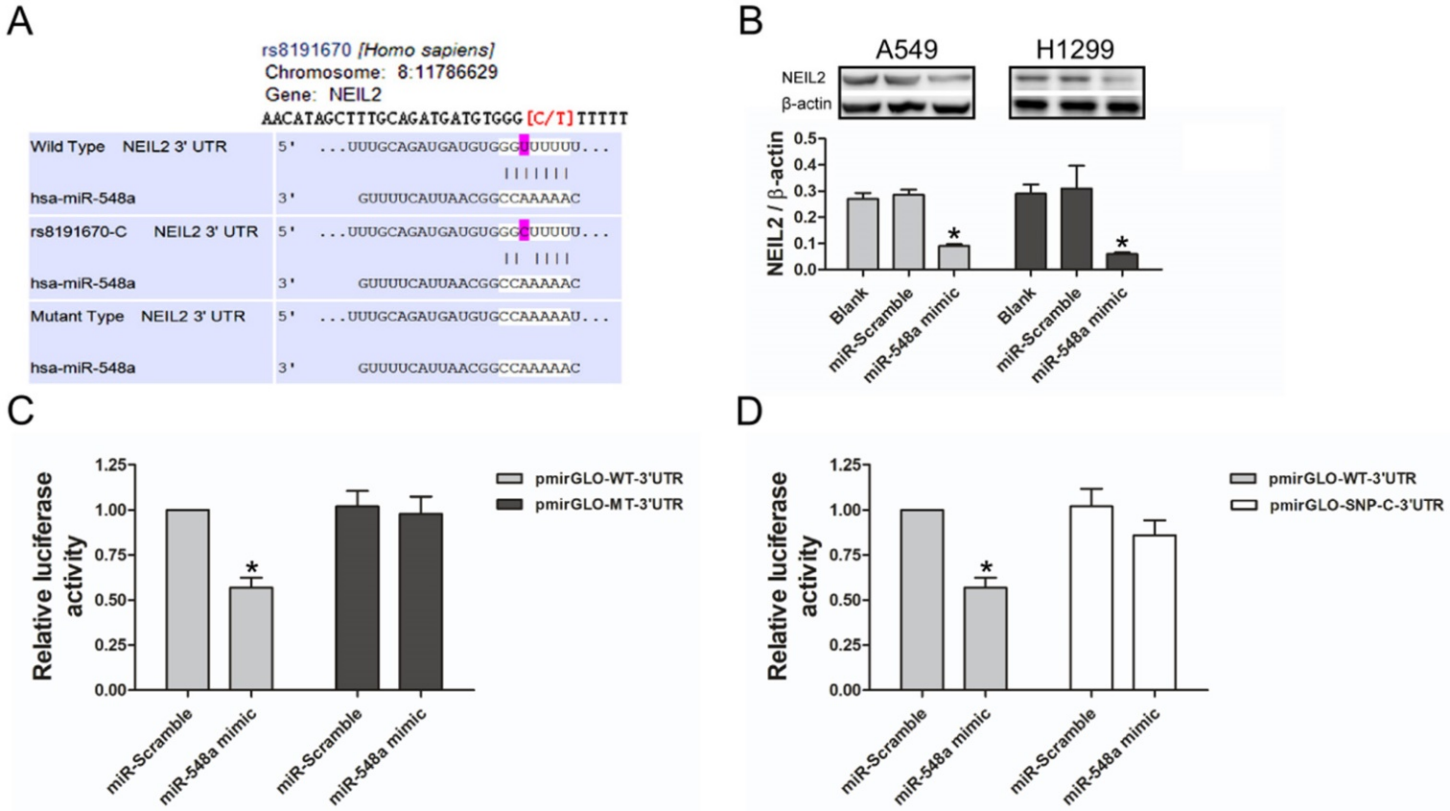
** MiR-548a targets *NEIL2* 3'UTR to suppress its expression. A:** Computational algorithms predicts that miR-548a might target *NEIL2* 3'UTR. **B:** MiR-548a mimic can significantly reduce the expression level of NEIL2 in both NSCLC cell lines. **C:** Compared with miR-Scramble, miR-548a mimic can significantly reduce the luciferase activity of pmirGLO-WT-3'UTR clone in HEK293T cells. However, neither miR-Scramble nor miR-548a mimic can affect the luciferase activity of mirGLO-MT-3'UTR clone. **D:** MiR-548a mimic cannot significantly reduce the luciferase activity of pmirGLO clone with SNP “C” in 3'UTR of *NEIL2* gene in HEK293T cells. *P<0.05.

To explore the potential regulating miRNA of *NEIL2* gene, a large number of target genes were predicted by three computational algorithms, which were TargetScan, miRanda, and PITA. The results showed that miR-548a might target *NEIL2* 3'UTR (Figure 2A). We then tested whether miR-548a can influence endogenous NEIL2 expression. Western blotting analysis showed that, both in A549 and H1299 cells, miR-548a mimic could induce a significantly reduction of endogenous NEIL2 expression (Figure 2B). In addition, 3'UTR luciferase reporter assays were preformed to verify whether miR-548a can bind to *NEIL2* 3'UTR and inhibit its expression. The result showed that miR-548a mimic significantly reduced the luciferase activity of wild type *NEIL2* clone (pmirGLO-WT-3'UTR), but it cannot affect the luciferase activity of mutant type *NEIL2* clone (pmirGLO-MT-3'UTR) (*P* < 0.05; Figure 2C). However, miR-scramble could not affect the luciferase activity of neither pmirGLO-WT-3'UTR nor pmirGLO-MT-3'UTR clone (*P* > 0.05; Figure 2C). These results provide the direct evidence that miR-548a can directly target the 3'UTR of *NEIL2* mRNA, resulting in translation suppression. But our results showed that miR-548a mimic could not reduce the luciferase activity of pmirGLO clone with SNP “C” in 3'UTR of *NEIL2* mRNA (*P* > 0.05; Figure 2D). Our results indicated that miR-548a can inhibit the translation of *NEIL2* mRNA by binding the seed region in its 3'UTR. When a single nucleotide “T” is replaced by “C” in *NEIL2* gene at rs8191670 locus, miR-548a cannot bind this seed region and lose the translational inhibition of *NEIL2* mRNA. These findings are also consistent with our clinical observation that the expression level of NEIL2 protein was significantly higher in NSCLC patients bearing “C/C” homozygous and “T/C” heterozygous than in “T/T” homozygote patients.

### Upregulation of NEIL2 expression could reduce the sensitivity of NSCLC cells to cisplatin

**Figure 3:**
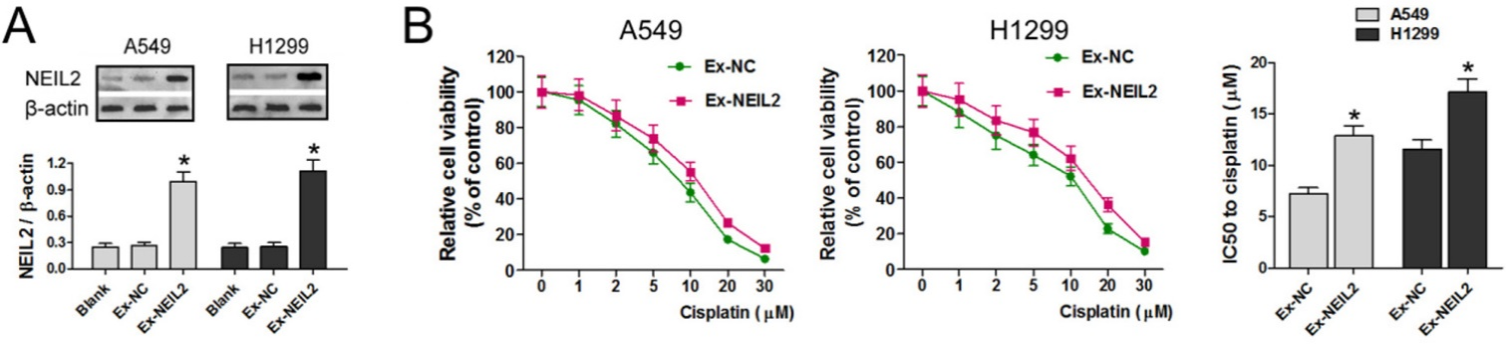
** Upregulation of NEIL2 expression could reduce the sensitivity of NSCLC cells to cisplatin. A:** NEIL2 is upregulated in NSCLC cells by exogenous expression. **B:** Upregulation of NEIL2 expression could increase IC50 value of NSCLC cells to cisplatin. EX: exogenous; NC: negative control. *P<0.05.

Western blotting assay was used to detect the expression of NEIL2 protein in both A549 and H1299 cell lines. Results displayed that exogenous *NEIL2* mRNA (Ex-NEIL2) successfully increased the expression of NEIL2 protein in both cell lines, compared with blank control and exogenous negative-control (Ex-NC). That is to say, NEIL2 protein was upregulated in NSCLC cells by exogenous expression with statistically significant difference (*P* < 0.05; Figure 3A).

To explore whether NEIL2 affects the sensitivity of NSCLC cells to cisplatin treatment, the cell viability was evaluated by MTT assay. The results showed that cisplatin treatment reduced the viability of A549 and H1299 cell lines cell in a dose dependent manner in both groups, but in Ex-NEIL2 group, the cell viability of both cell lines was increased in every dose of cisplatin. Our data showed that IC50 value of NSCLC cells to cisplatin treatment was significantly increased when NEIL2 expression level was upregulated (*P* < 0.05; Figure 3B), indicating that increased NEIL2 protein level can reduce the sensitivity of NSCLC cells to cisplatin.

### Downregulation of miR-548a could reduce the sensitivity of NSCLC cells to cisplatin by upregulating NEIL2 expression

**Figure 4:**
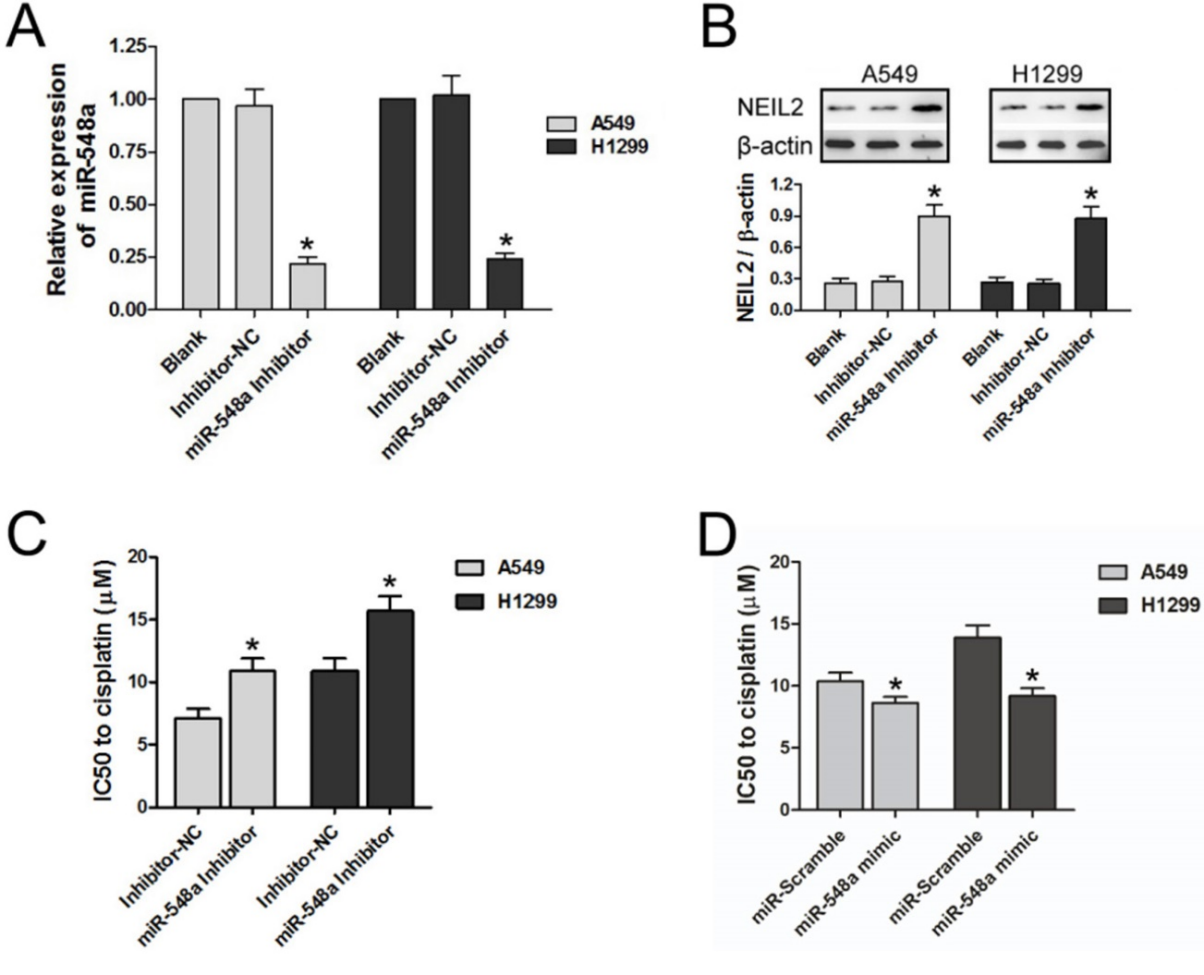
** Downregulation of miR-548a could reduce the sensitivity of NSCLC cells to cisplatin by upregulating NEIL2 expression. A:** miR-548a was downregulated by exogenous miR-548a inhibitor. **B:** Downregulation of miR-548a could increase NEIL2 expression in NSCLC cells. **C:** Downregulation of miR-548a could increase IC50 value of NSCLC cells to cisplatin. **D:** Upregulation of miR-548a could decrease IC50 value of NSCLC cells to cisplatin. NC: negative control. *P<0.05.

RT-PCR was tested to explore regulation of the exogenous miR-548a inhibitor on the expression of miR-548a in NSCLC cell lines. MiR-548a level was downregulated by exogenous miR-548a inhibitor in both A549 and H1299 cell lines, compared with the blank group and inhibitor negative control group (Inhibitor-NC) (*P* < 0.05; Figure 4A).

We then tested whether the miR-548a inhibitor can regulate NEIL2 expression. In both A549 and H1299 cells, NEIL2 protein expression level is significantly higher in the presence of miR-548a inhibitor than in the blank or inhibitor negative control group (Inhibitor-NC) (*P* < 0.05; Figure 4B).

We also tested the effect of miR-548a inhibitor on the sensitivity of NSCLC cells to cisplatin treatment. The IC50 values of both A549 and H1299 cells to cisplatin treatment were significantly increased in the presence of miR-548a inhibitor, compared to inhibitor negative control group (Inhibitor-NC) (*P* < 0.05; Figure 4C). On the contrary, miR-548a mimic could increase the sensitivity of NSCLC cells to cisplatin compared to miR-Scramble (*P* < 0.05; Figure 4D). Our results indicated that miR-548a can increase the sensitivity of NSCLC cells to cisplatin treatment through translational suppression of *NEIL2* mRNA.

## Discussion

Our study identified rs8191670, a single nucleotide polymorphism in *NEIL2* gene in NSCLC, revealed the role of NEIL2 in NSCLC chemoresistance and elucidated NEIL2 protein expression is regulated by miR-548a. *NEIL2* gene rs8191670 polymorphism has impact on mPFS of advanced NSCLC patients treated with cisplatin-based chemotherapy. Patients bearing “T/T” homozygote in *NEIL2* gene rs8191670 locus have better mPFS than bearing “C/C” homozygote. The underlying molecular mechanisms suggested that miR-548a regulates *NEIL2* mRNA translation by binding to its 3'UTR seed region containing rs8191670.

Some of studies were found that the mutation of *NEIL2* gene was associated with tumor development and progression. A recent study found that the SNP in the 5′- regulatory region of the *NEIL2* gene was associated with the risk of oral cancer 18. The CC genotype at the 4102971 locus of the *NEIL2* gene significantly increased the incidence of oral squamous cell carcinoma, especially advanced oral squamous cell carcinoma. Another study reported that genetic mutations in the coding region of the *NEIL2* gene correlated with the risk of rectal cancer 19. As has been found in colorectal cancer, *NEIL2* gene variants (R103Q, R103W, P123T, and R257L) are risk factors for colorectal cancer 20. Among these known SNPs of *NEIL2*, only R103Q and R257L are common in lung cancer, and the most frequent SNP is R257L 21. This particular study showed that the R257L mutant of *NEIL2* reduces the DNA glycosylase activity, weakens the ability of DNA damage repair, induces gene mutations, and eventually leads to lung cancer.

However, the polymorphism (T/C) in rs8191670 of *NEIL2* gene and its correlation with chemoresistance in NSCLC is largely unknown. Our study first identified the SNP (T/C) at rs8191670 locus of *NEIL2* gene and confirmed its association with the mPFS of advanced NSCLC patients receiving cisplatin based chemotherapy. Our clinical observations suggested that NEIL2 may be involved in chemoresistance of NSCLC and the expression level of NEIL2 was correlated with its polymorphism (T/C) in rs8191670.

To test our hypothesis, we performed a series *in vitro* analysis. First of all, we located rs8191670 at the 3'UTR of *NEIL2* mRNA, where miR-548a can bind with to regulate its translation (Figure 2A, 2B and 4A, 4B). MiRNAs binding to 3'UTR seed regions of targeting mRNA could lead to mRNA degradation or post-transcription inhibition, which in turn inhibits gene expression at post-transcription levels 22-23. Furthermore, other studies have documented that SNPs in the 3'UTR of the miRNA target gene [e.g., SNPs within miRNA (miRSNPs)] could interfere with the mRNA stability itself 24-25.

To explain how the expression level of NEIL2 was correlated with its polymorphism (T/C) in rs8191670, single nucleotide mutant (T/C in rs8191670) *NEIL2* 3'UTR reporter plasmid was constructed and luciferase assays were performed. Our results showed that miR-5487a can inhibit the translation of NEIL2 mRNA by binding with the seed region in its 3'UTR. But if “T” is replaced by “C” in *NEIL2* gene rs8191670 locus, miR-548a cannot bind to the seed region and lose the inhibition to NEIL2 mRNA translation (Figure 2C).

To date, there are only a few reports about the function of miR-548. One study provided evidences that miR-548k was over-expressed in esophageal squamous cell carcinoma (ESCC) and significantly associated with overall survival and lymph node metastasis. Their functional studies revealed oncogenic characteristics of miR-548k in ESCC aggressiveness. And the mechanistic studies demonstrate miR-548k target Weel, KLF10 and ADAMTS1 to promote cell cycle progression, proliferation, cell motility and lymphangiogesis 26. Heyn H 27 reported that miR-548d could induce apoptosis, cause cell cycle arrest, and inhibition of cell proliferation in pancreatic cancer. Overexpression of miR-548d could also enhance the sensitivity of pancreatic cancer cells to the gemcitabine. One study reported that the expression of mir-548-3p in breast cancer tissues was extremely low. Overexpression of mir-548-3p induced cell apoptosis and inhibited cell proliferation in breast cancer cells. The underlying molecular mechanism is that miR-548-3p binds to the 3'UTR of ECHS1 to inhibit the expression of ECHS1 28. Recently study revealed the expression profile and biological function of miR-548c-3p in osteosarcoma, which showed that decreased expression of miR-548c-3p in osteosarcoma contributed to cell proliferation via targeting ITGAV 29. In NSCLC, one report showed that miR-548b can inhibit proliferation and induce apoptosis of lung cancer cells by blocking PI3K/AKT signaling pathway 30.

Based on the previous studies mentioned above, to verify the role NEIL2 played in chemoresistance of NSCLC, exogenous expression of NEIL2 protein was introduced in order to upregulate the overall expression level of NEIL2 in NSCLC cell lines. In the presence of overexpressed NEIL2 protein, the sensitivity of NSCLC cells to cisplatin was significantly reduced (Figure 3). In the meantime, Downregulation of miR-548a reduces the sensitivity of NSCLC cells to cisplatin (Figure 4), indicating that miR-548a can affect the chemosensitivity of NSCLC cells through NEIL2.

In conclusion, our study demonstrated that *NEIL2* gene rs8191670 polymorphism affects PFS of advanced NSCLC patients receiving cisplatin-based chemotherapy. The underlying molecular mechanisms may be that miR-548a regulates *NEIL2* mRNA expression by binding to its 3'UTR seed region containing rs8191670. Our findings will be a strong indicator to predict the possible clinical outcomes of cisplatin treatment. The new “miR-548a - NEIL2” pathway warrants further investigations to overcome chemoresistance of NSCLC.

## Supplementary Material

Supplementary tables.
