# Supplementary material for: Nei Endonuclease VIII-like 2 Gene rs8191670 Polymorphism affects the Sensitivity of Non-small Cell Lung Cancer to Cisplatin by binding with MiR-548a
Source: J Cancer. 2020 Jun 6;11(16):4801–9. doi: 10.7150/jca.47495 (PMC7330683; doi:10.7150/jca.47495)
Supplement: Supplementary file 1 — Supplementary tables. [file jcav11p4801s1.pdf]

Table 1 The clinicopathological characteristics of non-small cell lung cancer (NSCLC) patients and the chemotherapy regimens (N=206)

**Characteristic**	**Case n (%)**
**Gender**	
Male	130(63.1)
Female	76(31.9)
**Age/year**	
≤60	101 (49.0)
>60	105(51)
**Smoking history**	
Yes	95 (46.1)
No	111 (53.9)
**Histological type**	
Adenocarcinoma	85 (41.3)
Squamous cell	121 (58.7)
**Chemotherapy regimen**	
Pemetrexed-platinum	85 (41.3)
Gemcitabine-platinum	112 (54.4)
Docetaxel-platinum	9 (4.3)

Table 2 The associations between the rs8191670 polymorphism of Nei endonuclease Ⅷ-like 2 (Neil 2) gene and the incidence rate of drug-related adverse reactions (N=206, n (%))

Toxicity	T/T type(n=110)		T/C type(n=54)		C/C type(n=42)		*P* value
	Grade 1-2	Graded≥3	Grade 1-2	Graded≥3	Grade 1-2	Graded≥3	
Nausea	50(45.5)	2(1.8)	21(38.9)	0(0.0)	18(42.9)	0(0.0)	0.21
Vomiting	30(27.3)	1(0.1)	18(33.3)	0(0.0)	14(33.3)	0(0.0)	0.17
Diarrhea	0(0.0)	0(0.0)	0(0.0)	0(0.0)	0(0.0)	0(0.0)	0.50
Leukopenia	62(56.4)	4(3.6)	24(44.4)	1(1.8)	20(47.6)	1(2.3)	0.35
Anemia	48(43.6)	1(0.1)	20(37.0)	0(0.0)	16(38.0)	0(0.0)	0.26
Thrombocytopenia	20(18.2)	2(1.8)	12(22.2)	1(1.8)	9(21.4)	0(0.0)	0.40
Fever related -leukopenia	0(0.0)	0(0.0)	0(0.0)	0(0.0)	0(0.0)	0(0.0)	0.50
